# The Essential Role of the *Deinococcus radiodurans ssb* Gene in Cell Survival and Radiation Tolerance

**DOI:** 10.1371/journal.pone.0071651

**Published:** 2013-08-09

**Authors:** J. Scott Lockhart, Linda C. DeVeaux

**Affiliations:** Department of Biological Sciences, Idaho State University, Pocatello, Idaho, United States of America; Florida International University, United States of America

## Abstract

Recent evidence has implicated single-stranded DNA-binding protein (SSB) expression level as an important factor in microbial radiation resistance. The genome of the extremely radiation resistant bacterium *Deinococcus radiodurans* contains genes for two SSB homologs: the homodimeric, canonical Ssb, encoded by the gene *ssb,* and a novel pentameric protein encoded by the gene *ddrB*. *ddrB* is highly induced upon exposure to radiation, and deletions result in decreased radiation-resistance, suggesting an integral role of the protein in the extreme resistance exhibited by this organism. Although expression of *ssb* is also induced after irradiation, Ssb is thought to be involved primarily in replication. In this study, we demonstrate that Ssb in *D. radiodurans* is essential for cell survival. The lethality of an *ssb* deletion cannot be complemented by providing *ddrB in trans*. In addition, the radiation-sensitive phenotype conferred by a *ddrB* deletion is not alleviated by providing *ssb in trans*. By altering expression of the *ssb* gene, we also show that lower levels of transcription are required for optimal growth than are necessary for high radiation resistance. When expression is reduced to that of *E. coli*, ionizing radiation resistance is similarly reduced. UV resistance is also decreased under low *ssb* transcript levels where growth is unimpaired. These results indicate that the expression of *ssb* is a key component of both normal cellular metabolism as well as pathways responsible for the high radiation tolerance of *D. radiodurans.*

## Introduction

The bacterium *Deinococcus radiodurans* is well known for its naturally high resistance to radiation and desiccation. Although much work has been done to elucidate the mechanisms that allow for this high tolerance, no single determinant has been identified. DNA and protein are both sensitive to radiation-induced damage [1], [2], and the high radiation tolerance of *D. radiodurans* is likely the result of a combination of efficient DNA damage repair and protein protection systems. *D. radiodurans* also contains between 4 and 10 copies of the genome at any given time, which may provide a reservoir of undamaged DNA for efficient, error-free repair [3]. In the context of DNA repair, single-stranded DNA-binding (SSB) proteins spatially and temporally coordinate activities of repair enzymes, and protect vulnerable single-stranded DNA (ssDNA) that is often produced during DNA repair as well as directly from damage [4], [5]. Recent evidence has implicated SSB expression levels as an important factor in radiation tolerance in many organisms. For example, in the eukaryotic fission yeast *Schizosaccharomyces pombe,* mutation or loss of Replication Protein A (RPA), the heterotrimeric SSB found in eukarya and archaea, results in DNA repair deficiencies and significantly increased sensitivity to radiation [6]. Interestingly, although eukaryotic RPA proteins are structurally distinct from bacterial SSB proteins [7], a conserved human SSB protein was recently identified that is more closely related to bacterial/crenarchaeal SSB proteins than its eukaryotic counterparts [8]. Reduced expression of this human protein resulted in increased sensitivity to ionizing radiation, but did not affect replication [8], [9].

The genome of *D. radiodurans* contains genes for two identified SSB proteins. The canonical-type protein, Ssb, encoded by the gene *ssb* (DR_0099-DR_0100) [10], forms a homodimer, with each subunit contributing two characteristic OB (oligonucleotide/oligosaccharide-binding) folds. In contrast, most bacterial SSBs form homotetramers, with each subunit contributing a single OB fold [5]; however, the crystal structures reveal conservation among the various bacterial SSB proteins [11]. Recent work on the ssDNA-binding states reveals a similarity between *D. radiodurans* Ssb and the well-studied homotetrameric *Escherichia coli* SSB, where both display two salt-dependent binding modes [12]. In *D. radiodurans*, levels of Ssb are ten to one hundred-fold higher, under non-stressed conditions (absence of radiation exposure), than levels of the homologous protein in the relatively radiation-sensitive *E. coli* [11]. Moreover, in *D. radiodurans* the expression of the *ssb* gene and the levels of Ssb protein increase four to six-fold after exposure to radiation [11], [13], whereas in *E. coli*, levels are unaffected by radiation exposure [14].

In addition to the canonical SSB, *D. radiodurans* produces an alternative single-stranded DNA binding protein, encoded by the gene *ddrB* (DR_0070), with a unique pentameric structure not yet found outside the Deinococcus/Thermus group [15]. This gene is expressed at very low levels in the absence of radiation, but is one of the most highly expressed genes following moderate doses of ionizing radiation [16], [17], indicative of a significant role in radiation damage recovery, where it is thought to facilitate RecA-independent repair [18]. The radiation tolerance of *D. radiodurans* is significantly reduced when *ddrB* is deleted, but it is not an essential gene [17]. The presence of two single-stranded DNA binding proteins in *D. radiodurans* suggests a division of function. Given the highly radiation-dependent expression of DdrB, the protein is likely to be involved specifically in radiation-induced damage, while the canonical Ssb is likely the major protein in replication. Although the *E. coli ssb* gene is essential for cell survival [14], this has not yet been demonstrated for the homologous gene in *D. radiodurans*.

It is clear that SSBs are important for both replication and DNA damage repair. However, it is unclear to what extent the expression levels of the various genes encoding SSBs contribute to the functionality of DNA repair systems and ultimately tolerance to DNA damaging agents such as radiation. In the haloarchaea, multiple RPA homologs have also been identified [19]. Mutants of the haloarchaeon *Halobacterium salinarum* ssp. NRC-1 that survive higher radiation doses than *D. radiodurans* exhibit constitutive expression of one operon encoding a radiation-induced RPA homolog, suggesting an important role of these proteins in extreme radiation resistance [20]. It is unknown what unique roles the multiple SSB proteins present in *D. radiodurans* play in the extreme resistance of the organism. Here we report that altered expression of the canonical-type SSB, encoded by the gene *ssb* in *D. radiodurans*, affects ionizing radiation tolerance, ultraviolet radiation tolerance, and general growth capabilities. We demonstrate that a much lower level of expression of the gene is sufficient for optimal growth than is necessary for high tolerance to ionizing and ultraviolet radiation; however, complete loss of *ssb* expression is lethal. In addition, we show that neither *ssb* nor *ddrB*, when constitutively expressed on a plasmid, complements a deletion of the other gene, demonstrating that the functions of the two single-stranded DNA binding proteins in *D. radiodurans* are distinct.

## Materials and Methods

### Strains and Growth Conditions

*D. radiodurans* strains (Table 1) were grown at 30°C in TGY (0.5% tryptone, 0.3% yeast extract, 0.1% glucose) broth or on TGY plates (1.5% agar) [21] for general manipulations. For transformations and inductions of plasmid-borne gene expression, 2× TGY was used. Chloramphenicol (3 µg mL^−1^) and streptomycin (8 µg mL^−1^) were added as appropriate. *Escherichia coli* (Table 1) strains were grown at 37°C in Luria-Bertani (LB) medium. Ampicillin (100 µg mL^−1^) or chloramphenicol (12.5 µg mL^−1^) was added as appropriate. *D. radiodurans* was transformed with approximately 1 µg of DNA as described by C. Bonacossa de Almeida, et al [22]. Cells were allowed to recover in 2× TGY for 5 hours for replicative plasmids and 18 hours for DNA requiring chromosomal integration, prior to plating on appropriate selective media. *E. coli* was transformed using CaCl_2_ chemical competence protocol and plated with appropriate selection [23].

**Table 1:** Strains and plasmids used in this study.

Strain or Plasmid	Description	Reference or Source
*D. radiodurans* GY10973	Wild-Type R1 *amyE* Ω (p*tufA*:*lacI kan^R^*)	[24]
*D. radiodurans* SL102	GY10973/pSL202 *ssb* Ω p*kat:aadA*	This Study
*D. radiodurans* SL101	GY10973 *ddrB* Ω p*kat:aadA*	This Study
*E. coli* DH5α	F- φ80*lacZ*ΔM15 Δ(*lacZYA-argF*) *U169 recA1 endA1 hsdR17* (rk-, mk+) *phoA supE44* λ- *thi-1 gyrA96 relA1*	Invitrogen
pCR4Blunt-TOPO	Plasmid for direct cloning of blunt-end PCR products utilizing topoisomerase I	Invitrogen
pSSBT	pCR4Blunt-TOPO::*ssb*	This Study
pDDRBT	pCR4Blunt-TOPO::*ddrB*	This Study
p11530	IPTG inducible expression vector for *D. radiodurans*	[24]
pSL201	p11530::*ddrB*	This Study
pSL202	p11530::*ssb*	This Study
pTNK103	pGEM-T::p*katA-aadA*	[17]

### DNA Manipulations

All primers are presented in Table 2. PCR reactions were performed using Phusion DNA polymerase (Finnzymes Lafayette, CO). Restriction enzymes were obtained from Fermentas (Glen Burnie, MA). Ligation reactions contained 10∶1 insert to vector using T4 DNA ligase (Promega, Madison, WI).

**Table 2:** Primers used in this study.

Primer	Sequence	Comment
ssbF	5′GCGCATATGGCCCGAGGCATGAACCA3′	*NdeI*
ssbR	5′GCGCTCGAGTTAAAAGGGCAGGTCGTC3′	*XhoI*
ddrBF	5′CGCGCATATGTGTTATGTTATTTACGTAAGGAGG3′	*NdeI*
ddrBR	5′GCGCTCGAGTCAGAACGGCGTTTCTTCT3′	*XhoI*
strepF	5′ATTTGTTATGGCCCGCGAGGGCCTGAGGGCCATG3′	
strepR	5′CCCAGGGCCTGCATTAAAATCGAAAGTTTAAACTTATTTGCCGACTACC3′	
ssbUpF	5′AGCGCGGGGGGCCTGACCC3′	
ssbUpR	5′CCCTCGCGGGCCATAACAAATTCTCCTTGGGTAGCTG3′	
ssbDwnF	5′GCAAATAAGTTTAAACTTTCGATTTTAATGCAGGCCCTGGGGGCC3′	
ssbDwnR	5′TCGGGGTGGGCGCGGTAAGCGAT3′	
ddrBUpF	5′CTGCGGCCTTACCTCAACACCTTCTGG3′	
ddrBDwnR	5′GCACCTCGTACTGCTTGACGTTCTCGC3′	
ssbConfUpF	5′GCAAGGGCCGTCCGCCAGCAACAT3′	
ssbConfDwnR	5′GCTGGGTCATGTTGGGTGTCCTTGGTG3′	
strepIntF	5′TTCGGCTTCCCCTGGAGAGAGCGAGA3′	
strepIntR	5′TCTCGCTCTCTCCAGGGGAAGCCGAA3′	
ddrBConfUpF	5′CTGAAGGTCTTGGTGCCCGGCCTCT3′	
ddrBConfDwnR	5′ATACGCAACACGAAAGCAGGCCGCCTTC3′	
11530seqF	5′CGTTTCCGGGGGTCAGCTTCCCAG3′	
11530seqR	5′CGGCGGGCAGTGAGAGATCCGAGAAA3′	
qGAP F	5′ATCAACATCATTCCCACCTCG3′	
qGAP R	5′TACCACGAGAAGAACTTGACGA3′	
qSSB F	5′TGGTTGAAGGTACCCTGGAATA3′	
qSSB R	5′GGCGTCGATATAGTGAACCTTT3′	

*D. radiodurans* genomic DNA was prepared from 5 mL cultures following centrifugation and resuspension in 1 mL of 95% ethanol. After a ten minute incubation at room temperature, the suspensions were centrifuged and resuspended in 100 µL of 5 mg mL^−1^ lysozyme in TE pH 8.0 and incubated at 37°C for 30 minutes. Following incubation, 500 µL of 2% SDS, 0.1 M Na_2_EDTA, 0.1 mg mL^−1^ proteinase K was added and the solution incubated 30 minutes at 50°C. The solution was extracted with 25∶24∶1 phenol/chloroform/isoamyl alcohol, then treated with RNAse (7 units) at 37°C for 30 minutes. The solution was re-extracted, and DNA was precipitated with ethanol.

Plasmid DNA was isolated from 1.5 mL of *D. radiodurans* culture following centrifugation and resuspension in 100 µL of 25 mM Tris-HCl pH 8.0, 10 mM Na_2_EDTA, 50 mM glucose, 2 mg mL^−1^ lysozyme, 5 µg mL^−1^ proteinase K. The solution was incubated at 50°C for 30 minutes, 0°C for 5 minutes and finally at 95°C for 1 minute. After incubation, 200 µL of 1% SDS, 0.2 M NaOH was added and gently mixed, followed by gentle mixing with 150 µL of 3 M Na-acetate. The lysed cell solution was centrifuged to remove debris. The supernatant was decanted and treated with RNAse (7 units) at 37°C for 30 minutes, followed by extraction with phenol/chloroform/isoamyl alcohol, and ethanol precipitation. Plasmid DNA from *E. coli* was prepared using the StrataPrep (Stratagene, Santa Clara, CA) plasmid mini-prep kit according to the manufacturer’s instructions.

### Construction and Induction of *ssb* and *ddrB* Expression Plasmids

All plasmids are listed in Table 1. The *ssb* gene (DR_0099-DR_0100) was PCR amplified from *D. radiodurans* template genomic DNA using the primers ssbF/ssbR, which introduced *Nde*I and *Xho*I restriction endonuclease recognition sequences into the ends of the amplicon. The *ddrB* gene (DR_0070) was PCR amplified from *D. radiodurans* template using the primers ddrBF/ddrBR, which introduced *Nde*I and *Xho*I restriction endonuclease recognition sequences into the ends of the amplicon. The products were cloned into the pCR4Blunt-TOPO vector (Invitrogen, Grand Island, NY) producing pSSBT and pDDRBT, respectively, which were propagated in *E. coli* DH5α. pSSBT and pDDRBT were each digested with *Nde*I and *Xho*I, and treated with shrimp alkaline phosphatase for 30 minutes at 37°C. The vector p11530 [24] was digested with *Nde*I and *Xho*I, extracted with phenol/chloroform/isoamyl alcohol, and precipitated with ethanol. Each fragment was ligated to digested p11530 vector, creating pSL202 (*ssb*) and pSL201 (*ddrB*). Constructions were verified by sequencing the entire cloned gene and flanking region of p11530 on an ABI 3130XL Genetic Analyzer at the Idaho State University Molecular Research Core Facility (primers 11530seqF/11530seqR). *D. radiodurans* strain GY10973, which contains the *E. coli lacI* gene inserted in the chromosome for control of the plasmid-borne inducible *spac* promoter [24], was separately transformed with the verified plasmids and uptake was confirmed by PCR.

Gene expression from pSL201 and pSL202 was induced in *D. radiodurans* as described by Lecointe et al. [24]. Growing cells were sub-cultured to an OD_600_ of approximately 0.05 and allowed to grow with shaking for 4 hours. IPTG was then added to the required final concentration (0–1 mM) and the cultures were grown an additional 12 to 14 hours before experimental treatments.

### RNA Extraction and Transcription Analysis

Fifty mL cultures of *D. radiodurans* were pelleted and re-suspended in 1.5 mL of lysis solution from a Total RNA Isolation Mini Kit (Agilent, Santa Clara, CA) with approximately 200 µL of 0.5 mm glass beads. The solution was homogenized in a Mini Beadbeater (Biospec Products, Bartlesville, OK) for 5 minutes. After centrifugation, the RNA was purified according to kit instructions. RNA quantity and quality were assessed using an Agilent 2100 Bioanalyzer with the RNA 6000 Nano kit. Superscript III Reverse Transcriptase (Invitrogen, Santa Clara, CA) was used to produce cDNA from RNA (10 µg). The cDNA product was purified with the Nucleospin II (Macherey-Nagel, Bethlehem, PA) reaction cleanup kit according to manufacturer instructions.

Semi-quantitative PCR was performed as previously described [25]. Primers qSSBF/qSSBR were used to analyze *ssb* expression. The housekeeping gene glyceraldehyde 3-phophate dehydrogenase (GAPDH) was also analyzed, using primers qGAPF/qGAPR, for normalization [17]. Samples were separated on a 1% agarose TAE gel, stained with ethidium bromide, and imaged using the Versadoc Gel Imaging System (BioRad, Hercules, CA) under parameters that disallowed signal saturation at any point in the image. Log-transformed band intensities were plotted against cycle number and the linear portions of these plots were fit using a standard linear regression (R^2^≥0.90) to determine relative expression. *ssb* levels were normalized to GAPDH.

### Construction of *ssb* and *ddrB* Deletion Mutants

The *ssb* and *ddrB* genes of *D. radiodurans* were deleted using a previously described gene-replacement system [17]. The target genes (DR_0099-DR_0100 and DR_0070) were individually replaced, via homologous recombination, using an engineered gene knockout fragment consisting of a streptomycin antibiotic resistance cassette flanked by the up- and down-stream sequence (750 bp) of the chromosomal gene. For deletion of the entire coding region of the *ssb* gene, the streptomycin antibiotic resistance cassette was amplified from pTNK103 [17] with primers strepF/strepR and the up- and down- stream flanking regions of the *ssb* gene were amplified from *D. radiodurans* genomic DNA using primers ssbUpF/ssbUpR and ssbDwnF/ssbDwnR. The *ssb* replacement fragment was assembled from these individual components using overlap-extension PCR [17], [26].

The *ddrB* gene had previously been deleted in *D. radiodurans* by Tanaka et al. (strain TNK122) [17]. To create a similar deletion mutant in strain GY10973, which contains the *E. coli lacI* gene for repression of the inducible *spac* promoter of the expression vector p11530, genomic DNA from TNK122 was PCR amplified using primers ddrBUpF/ddrBUPR. This produced a complete *ddrB* replacement fragment consisting of the streptomycin antibiotic resistance cassette flanked by the up- and down-stream regions of the *ddrB* gene.

Competent *D. radiodurans* was separately transformed with each gene replacement fragment. Genomic DNA was extracted from transformant colonies and screened via PCR for gene replacement using the appropriate primer sets. The primers used to confirm *ssb* replacement were ssbConfUpF/strepIntR, strepIntF/ssbConfDwnR, ssbF/ssbR and strepF/strepR, andfor *ddrB*: ddrBConfUpF/strepIntr, strepIntF/ddrBConfDwnR, ddrBF/ddrBR and strepF/strepR.

### Growth Curve Modeling

The OD_600_ measurements of the cultures were plotted versus time and fitted with a modified Gompertz growth function (Eq. 1) [27] using the *nls* nonlinear least squares curve-fitting function from R 2.13.1 [28].
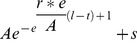
(1)


In Equation 1, “A” denotes the maximal OD_600_, “s” the starting OD_600_, “r” the maximum achieved growth rate, “l” the growth lag time, and “t” the total growth time.

### Ionizing Radiation Survival

Irradiations were performed at the Idaho Accelerator Center (Pocatello, ID) using a medical grade S-band LINAC, which delivered 23 MeV electrons in a 2 µs pulse at 60 Hz. We have previously demonstrated that irradiation under these conditions results in comparable survival to that of gamma irradiation from a ^60^Co source, for both *D. radiodurans* and the unrelated haloarchaeon *Halobacterium salinarum* sp. NRC-1 [20], [29]. Cultures were irradiated at room temperature in polypropylene PCR tubes, which were placed in electrically conductive plastic sample holders in a circular configuration. Sample holders were placed 80 cm from the beam port, where uniformity of the beam was within 10% of the peak dose over the area encompassing the sample holder. Samples were located on isocontours of dose with sample dose variations less than 1%. Beam profile and intensity was determined with NIST-traceable radiochromic film and a real-time Faraday cup dosimetry array [30]. Beam location was verified before and after irradiations. The delivered dose was measured with GEX B3 radiochromic film using a GEX Corporation (Centennial, CO) thin film dosimetry system.

For each strain irradiated, multiple experiments (independent cultures) were performed on at least two separate days. Expression plasmids were induced as necessary and aliquots of grown cultures (200 µL) were placed into polypropylene PCR tubes, and kept on ice until irradiation. Immediately after irradiation, the aliquots were serially diluted in growth medium and plated in duplicate. In each experiment, an unirradiated aliquot was similarly diluted and plated at the beginning and end of each experiment.

The viable cell density of a sample culture was calculated from counts of colony forming units (CFU) after 3 to 4 days incubation. The average CFU mL^−1^ of unirradiated sample aliquots represented 100% survival for a culture. The surviving fraction at a given dose was the average CFU mL^−1^ from each irradiated sample divided by that of the unirradiated sample.

To model the survival of each strain, surviving fractions from all pertinent experiments were combined and plotted against dose. The data was fitted with a Boltzmann logistic survival function (Eq. 2) [20], [31] using the *nls* non-linear least squares fitting function from R 2.13.1 [28].
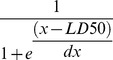
(2)


In Equation 2, horizontal asymptotes at 1 and 0 correspond to 100% and 0% survival, respectively. “LD_50_” is the inflection point of the curve, which occurs at the 50% survival point, and the term “dx” defines the width of the curve.

### Ultraviolet Radiation Tolerance Assay

Ultraviolet (UV) irradiations were performed using a UV-C lamp (254 nm). For each strain, multiple experiments (independent cultures) were performed on at least two separate days. Fluence was checked before and after each plate was exposed using a MS-100 optical radiometer with a MS 125 UVC sensor (Ultra-Violet Products, Upland, CA). Survival was modeled as for ionizing radiation exposure.

### Statistical Analyses

Parameters from growth, survival or expression (linear) models were compared among strains by calculating t-test p-values for all pair-wise strain comparisons using parameter estimates and standard errors generated by the least squares fits of the data. The pair-wise p-values were then adjusted to account for simultaneous inference using the modified Bonferonni algorithm for p-value adjustment by Holm [28], [32]. All comparisons were made at a significance level of α = 0.01. Due to the very large number of data points obtained for growth and survival modeling, graphical representations contain binned data points to allow for clearer presentation and interpretation. However, presented curves and parameter estimates were generated using the full datasets.

## Results

### The *ssb* Gene is Essential for Survival of *D. radiodurans*

SSBs are central components of DNA replication and repair pathways; thus, it was expected that altering the natural expression of the *ssb* gene in *D. radiodurans* would affect these pathways. To investigate these effects, the entire coding region of the *ssb* gene of *D. radiodurans* was replaced with an engineered deletion allele containing the streptomycin resistance gene, *aadA* [17]. Many attempts were made to complete this knockout in strain GY10973, which differs from the wild type strain R1 by carrying the *E. coli lacI* gene. Although gene replacements in naturally competent *D. radiodurans* are easily selected after transformation with a linear fragment containing an antibiotic resistance gene, because *D. radiodurans* is multigenomic, homozygosity must be confirmed through PCR [3]. Although streptomycin-resistant transformants were obtained, all of the transformants also carried the intact *ssb* gene. The likely explanation is that these cells were heterozygous at the *ssb* locus, suggesting that loss of *ssb* is lethal. To circumvent this, the transformation was repeated in strain GY10973/pSL202, which carries an IPTG-inducible copy of *ssb* on plasmid pSL202. Transformants were grown in the presence of IPTG, to induce expression of the plasmid-borne *ssb,* and streptomycin, to select for chromosomal integration of the knockout fragment. Streptomycin-resistant transformants were screened via PCR. Under these conditions, transformants were readily obtained in which *ssb* had been replaced in all copies of the genome. One of these PCR-verified mutants, which contained a chromosomal Δ*ssb* complemented *in trans* by plasmid-borne *ssb*, was designated strain SL102.

With *ssb* provided *in trans*, chromosomal *ssb* knockouts were obtained at a rate of 0.80 (80% of streptomycin resistant transformants did not contain a chromosomal copy of *ssb* while the remaining 20% did, as determined using PCR). Based on this empirically determined success rate for chromosomal *ssb* knockout, and the Wilson method for estimation of the binomial parameter [33], the true success rate was estimated to be 0.714 with bounds on the 99.9% confidence interval of 0.317 and 1.00. With this estimate, without *ssb* provided *in trans*, screening at least 18 streptomycin-resistant transformants for replacement of *ssb* by *aadA* in all copies of the genome would allow for determination of the essentiality of *ssb* at a confidence level of α = 10^−9^. Furthermore, at the conservative lower bound of the 99.9% confidence interval (0.317) essentiality could still be determined at a confidence level of α = .001.

To establish the essentiality of *ssb* with statistical certainty, GY10973 was again transformed with the *ssb* replacement fragment. Transformant colonies in excess of the required 18 (23 total) were randomly picked for screening. Diagnostic PCRs confirmed that all analyzed transformants were heterozygous, and contained the streptomycin-resistance gene, *aadA,* as well as intact *ssb*. The probability of this occurring without *ssb* being essential was less than.001 with at least 99.9% confidence. Thus, *ssb* was determined to be essential for survival of *D. radiodurans.*

### *ssb* Expression Levels are an Important Factor for Growth

Because SSBs play a central role in DNA replication, we used strain SL102, where the only copy of *ssb* was controllable by IPTG, to measure the effect of altered *ssb* expression on growth capabilities. Parallel growth studies were performed with GY10973/p11530 (*ssb^+^*/empty expression vector), GY10973/pSL202 (*ssb^+^*/*ssb* expression plasmid), and SL102 (Δ*ssb/ssb* expression plasmid). Since there were no differences in cell size and morphology between these strains when compared by microscopy (data not shown), it was assumed that cell density corresponded to OD_600_ similarly for all strains.

**Figure 1:**
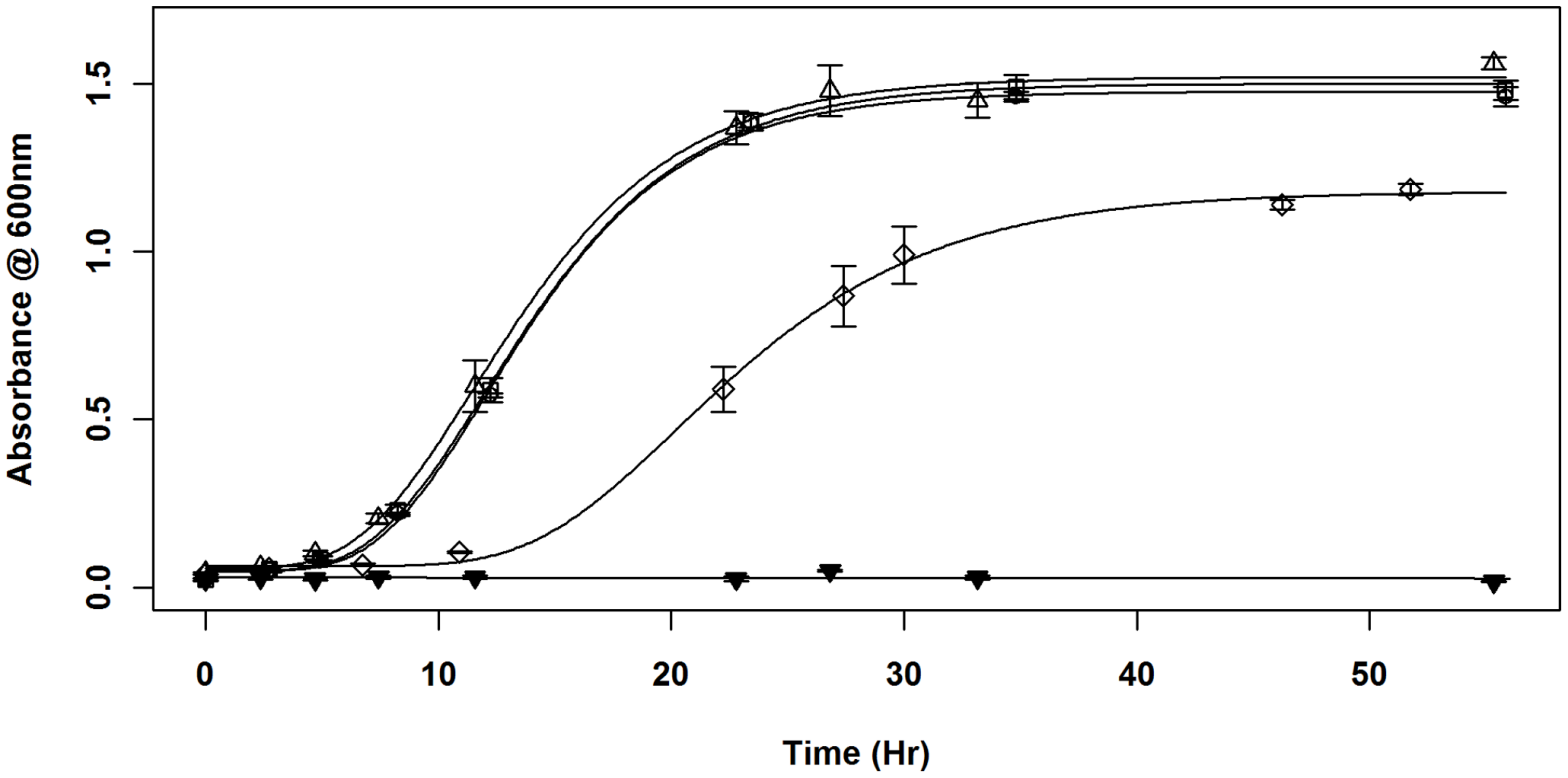
Effect of *ssb* expression on growth. Cultures initially grown in 1 mM IPTG were sub-cultured twice in either 1 mM, 0.04 mM or 0 mM IPTG. Growth in the second cycle is shown. SL102 (Δ*ssb/ssb* expression plasmid) in 1 mM (Δ), 0.04 mM (◊) and 0 mM (▾) IPTG. Since there was no difference in growth with or without IPTG of strains GY10973/p11530 (*ssb^+^*/empty vector) (○) and GY10973/pSL202 (*ssb^+^*/*ssb* expression plasmid) (□), only 0 mM growth is presented. Error bars indicate ± standard error.

**Figure 2:**
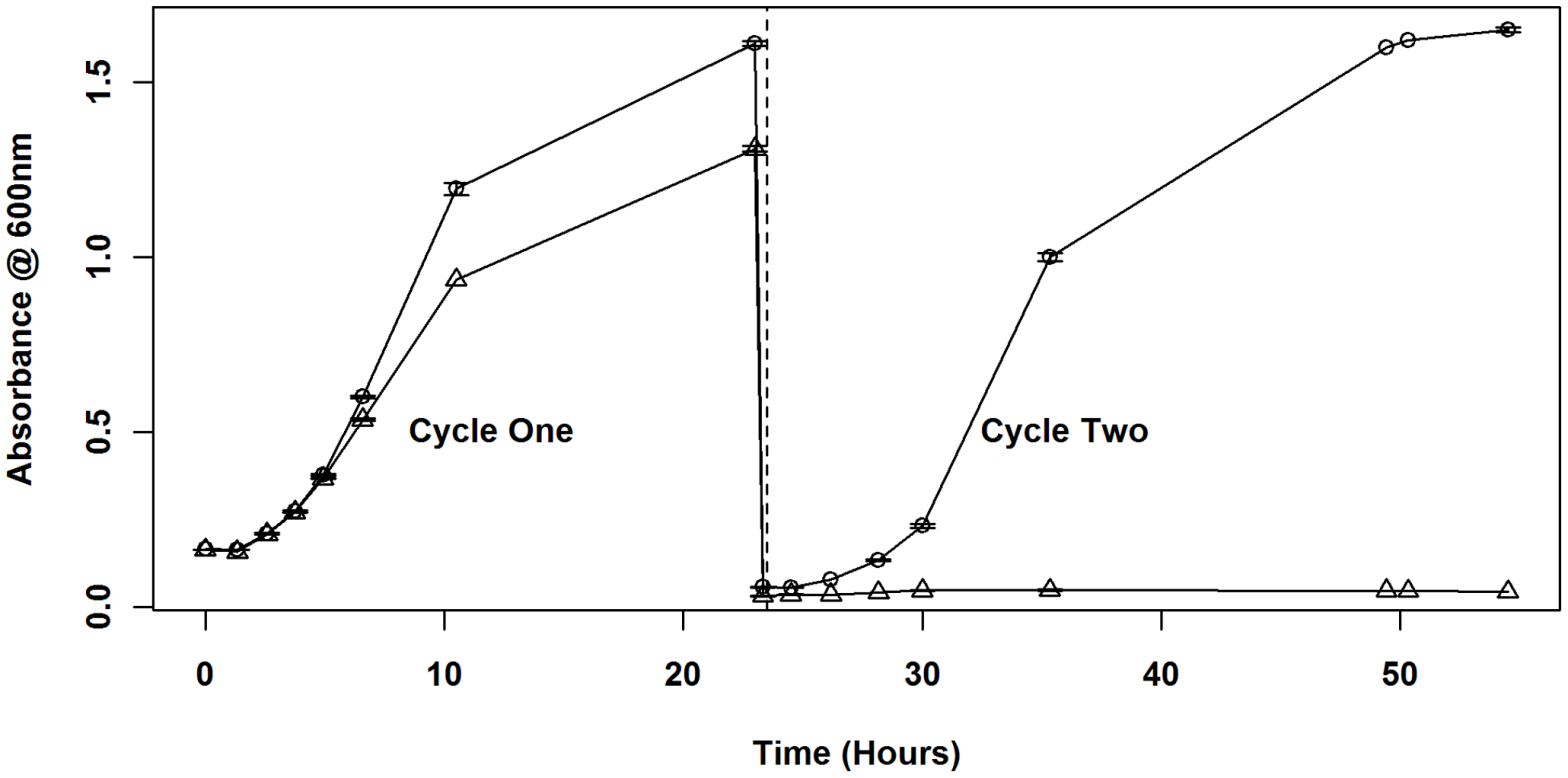
Effect of *ssb* depletion on growth. SL102 was initially grown in 1 mM IPTG, then sub-cultured twice in either 1 mM IPTG (○) or 0 mM IPTG (Δ). Error bars indicate ± standard error.

For these experiments, cultures initially grown under full induction (1 mM IPTG) were sub-cultured twice in three different concentrations of inducer: 1 mM IPTG, 0.04 mM IPTG or no IPTG. For the first cycle of growth, saturated cultures were diluted to an OD_600_ of ∼ 0.05, and allowed to grow 24 hours to ensure that any effects of the change in inducer concentration had equilibrated. These cultures were again sub-cultured to an OD_600_ of ∼ 0.05, for the second growth cycle, in the same IPTG concentration as the first cycle. In 1 mM IPTG (full induction) during both growth cycles, all strains had equivalent maximal growth rates and identical growth curves (p>0.01; Figure 1). This indicated that fully-induced *ssb* expression levels from the plasmid promoter in SL102 were sufficient to effectively carry out general replicative growth functions. However, reducing the concentration of IPTG in the medium to 0.04 mM reduced the maximal growth rate of SL102 to 53.0% of that observed under fully induced conditions, indicating that growth capabilities are dependent on *ssb* expression levels. When the IPTG was omitted from the medium, growth rates for strains GY10973/p11530 and GY10973/pSL202 were identical to those observed under conditions of 1 mM IPTG. However, the growth of SL102 slowed during the first cycle and stopped in the second cycle of growth, suggesting a loss of Ssb from the cells (Figure 2). These results suggest that cellular survival is dependent on the presence of Ssb. Combined with the inability to obtain complete *ssb* replacements without complementation *in trans,* these results confirmed that expression of *ssb* was essential for cell survival.

### Fully IPTG-induced *ssb* Expression is Significantly Less than Wild Type

**Figure 3:**
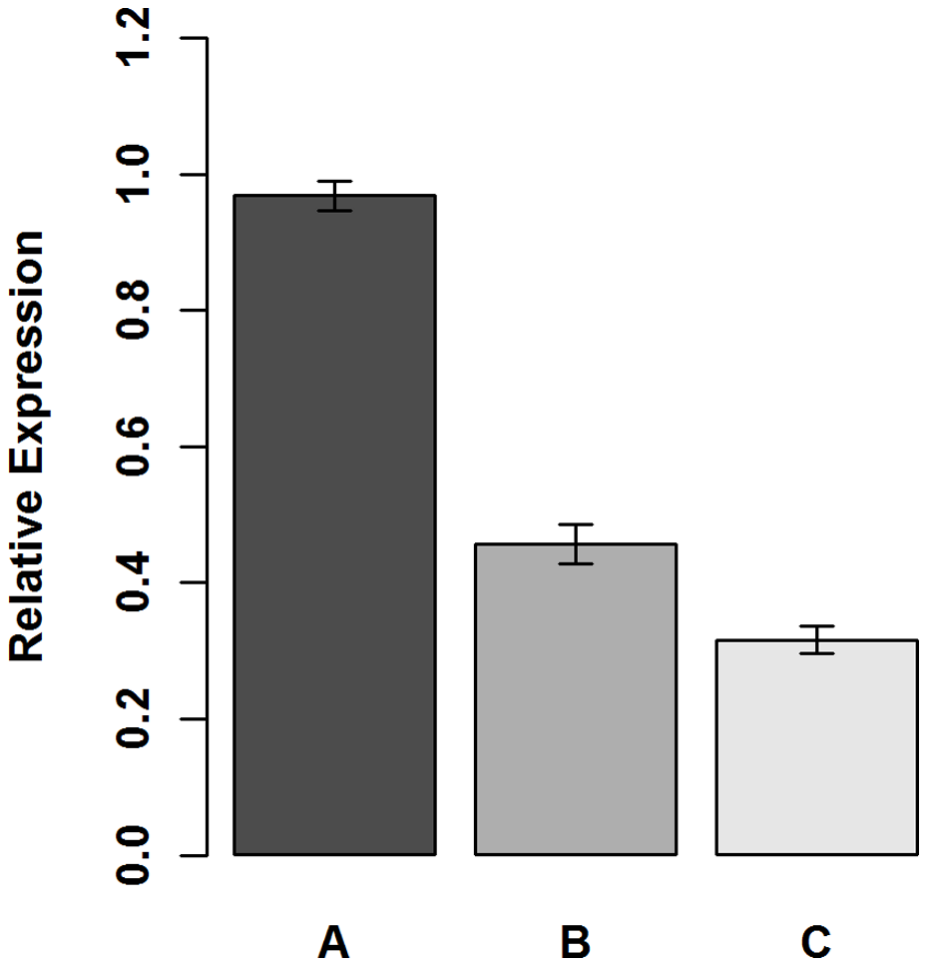
Relative *ssb* transcript levels. Semi-quantitative PCR was used to compare *ssb* expression between strains. Transcript levels of *ssb* in (A) GY10973/p11530 (*ssb^+^*/empty vector) grown in 1 mM IPTG, (B) SL102 (Δ*ssb/ssb* expression plasmid) grown in 1 mM IPTG and (C) SL102 grown in 0.04 mM IPTG are shown relative to *ssb* transcript levels in strain GY10973/pSL202 (*ssb* ^+^/*ss*b expression plasmid) grown in 1 mM IPTG. All *ssb* levels were normalized with GAPDH levels. Error bars indicate ± standard error.

To quantify *ssb* expression levels, semi-quantitative PCR was performed [25]. In strain SL102 (Δ*ssb/ssb* expression plasmid), the only *ssb* was under control of induction by IPTG. Strains GY10973/p11530 (*ssb^+^*/empty expression vector) and GY10973/pSL202 (*ssb^+^*/*ssb* expression plasmid) both carry wild type *ssb* in the chromosome. *ssb* expression in all three strains was examined under full inducing conditions (1 mM IPTG). In addition, expression in strain SL102 was examined using partially induced conditions (0.04 mM), in which growth was impaired. Data was normalized using the housekeeping gene GAPDH [17] and *ssb* transcript comparisons were made using linear model estimates at cycle number 26.5. *ssb* transcript level in strain GY10973/pSL202, the *ssb+* parent of strain SL102, grown in 1 mM IPTG was arbitrarily designated 100%. Absence of the inducible copy in GY10973/p11530 did not significantly affect *ssb* levels (Figure 3; p>0.01). However, under fully induced conditions, the expression of *ssb* in SL102 was only 42.0% of the expression level of the parent strain GY10973/pSL202 (Figure 3; p<0.01). Reduction of the inducing IPTG concentration to levels that impaired growth (0.04 mM) further decreased *ssb* expression in SL102 to 28.5% of GY10973/pSL202 expression (Figure 3; p<0.01). These results indicate that expression of *ssb* from the native promoter, even in the absence of radiation, is far higher than that from the fully induced promoter of the expression plasmid. Furthermore, although we could not significantly increase *ssb* expression levels in unstressed, wild type cells by providing an inducible, plasmid-borne copy of *ssb*, we significantly and controllably reduced *ssb* levels in SL102. This lower level of expression was sufficient to allow optimal growth; however, further reduction impaired growth.

### Altered *ssb* Expression Significantly Affects Ionizing Radiation (IR) Tolerance

The expression levels of SSB genes are important determinants for radiation resistance [20]. To determine the effects of altered expression of *ssb* on the ionizing radiation tolerance of *D. radiodurans*, the survival of strains GY10973/p11530 (*ssb^+^*/empty expression vector), GY10973/pSL202 (*ssb^+^*/*ssb* expression plasmid), and SL102 (Δ*ssb/ssb* expression plasmid) after irradiation with 23 MeV electrons from a linear accelerator was compared [20].

**Figure 4:**
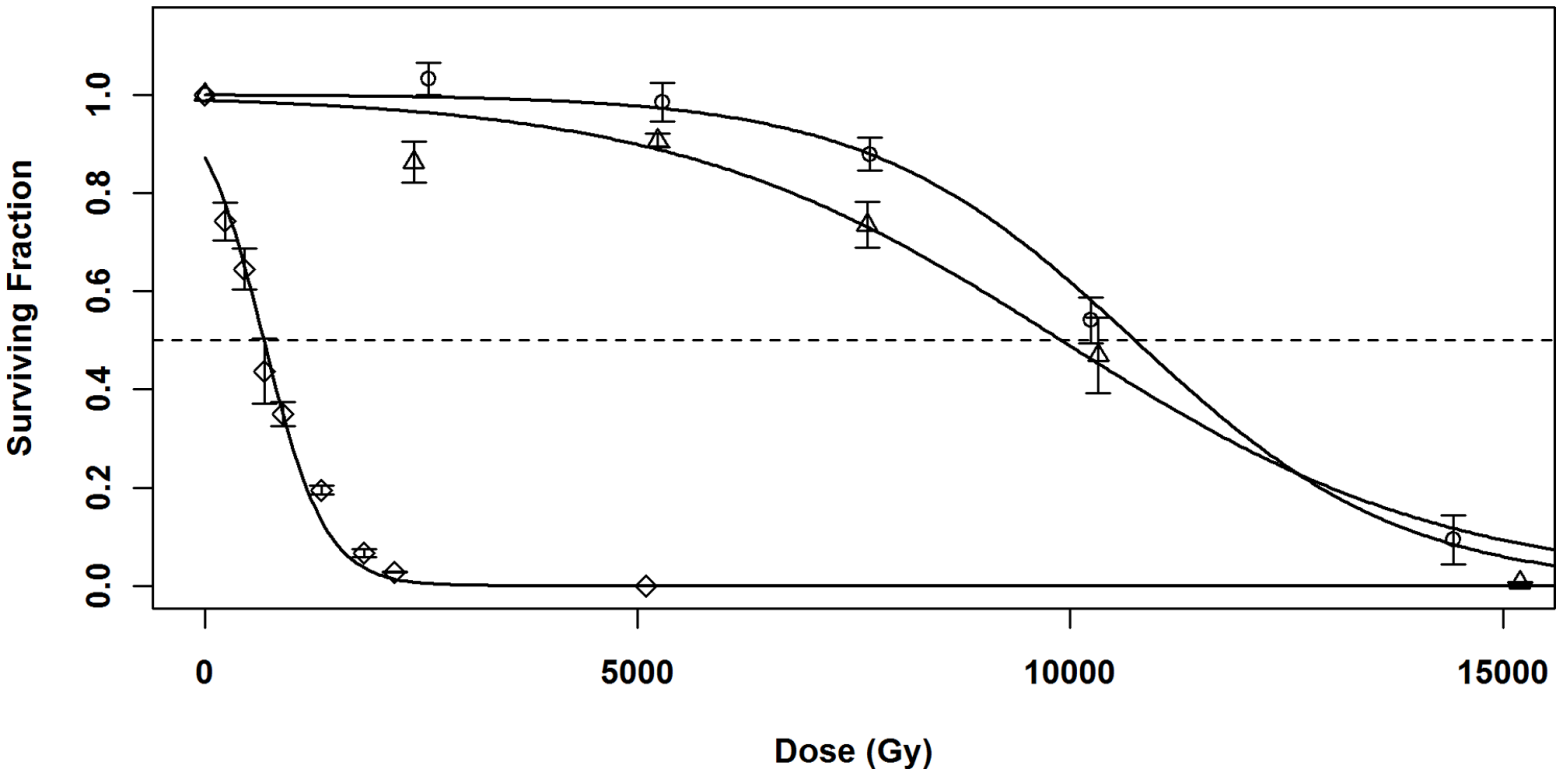
Ionizing radiation survival of strains with altered *ssb* levels. Cultures were grown in 1 mM IPTG and irradiated with 23 MeV electrons at a dose rate of 30 Gy/s. (○) GY10973/p11530 (*ssb^+^*/empty vector); (Δ) GY10973/pSL202 (*ssb^+^/ssb* expression plasmid) and (◊) SL102 (Δ*ssb/ssb* expression plasmid). Appropriate dilutions were plated, and survivors counted after 3–5 days. The dashed line indicates 50% survival. Error bars indicate ± standard error.

To determine if additional *ssb* expression increased ionizing radiation tolerance, GY10973/p11530 and GY10973/pSL202 were irradiated under full inducing conditions (1 mM IPTG). The LD_50_ values for GY10973/p11530 and GY10973/pSL202 were 10750 Gy (±207) and 9898 Gy (±419), respectively (Figure 4). Statistical analysis showed that these values were equivalent (p>0.01), indicating that providing a plasmid-borne gene had no significant effect on the radiation tolerance of *D. radiodurans.* In contrast, decreased *ssb* expression had a dramatic effect. Strain SL102 (Δ*ssb/ssb* expression plasmid) was also irradiated under full inducing conditions, which had provided enough *ssb* expression to allow growth (see Figure 1). Despite the normal growth phenotype, the LD_50_ of SL102 was 681 Gy (±35), which is only 6.9% (p<0.01) of the GY10973/pSL202 LD_50_ of 9898 Gy (±419) (Figure 4). Thus, reduced *ssb* expression, despite no deleterious effect on growth, resulted in severe deficiency of the ionizing radiation tolerance of *D. radiodurans.*

### Altered Expression of *ssb* Significantly Affects UV-C Radiation Tolerance

Ultraviolet radiation, like ionizing radiation, can cause lethal damage to DNA and other cellular structures. To determine the effects of altered *ssb* expression on the UV-C radiation tolerance of *D. radiodurans*, cultures grown in 1 mM IPTG were diluted and aliquots spotted onto plates prior to exposure to 254 nm UV-C at doses ranging from 0 J•m^−2^ to 1 kJ•m^−2^.

**Figure 5:**
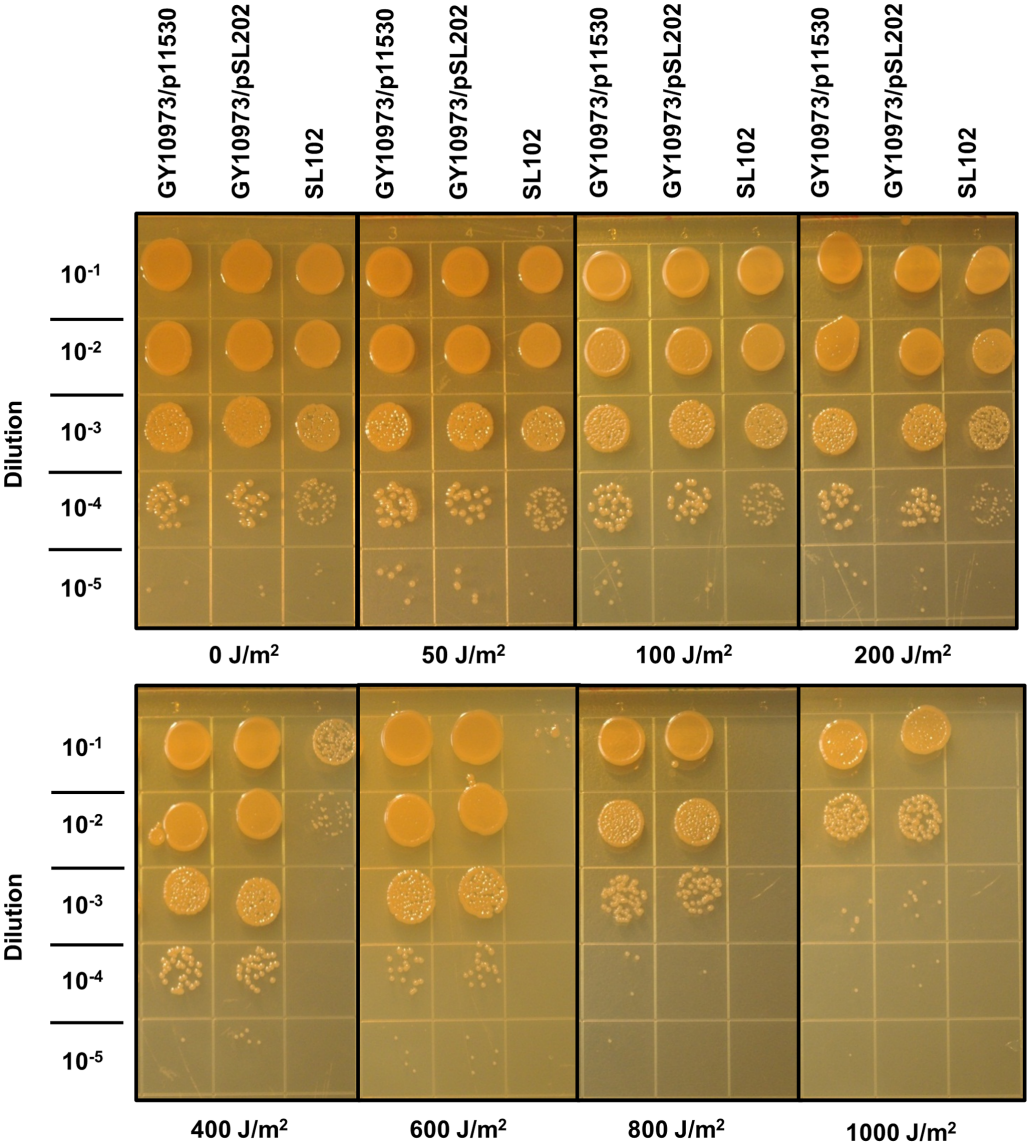
UV survival of strains with altered *ssb* levels. GY10973/p11530 (*ssb^+^*/empty vector), GY10973/pSL202 (*ssb^+^/ssb* expression plasmid) and SL102 (Δ*ssb/ssb* expression plasmid) were grown under full inducing conditions. Cultures were diluted and aliquots spotted onto plates and allowed to dry prior to exposure to UV-C. Surviving colonies were counted and used to determine surviving fractions, as described.

As with the ionizing radiation tolerance assays, providing *ssb* on the plasmid did not increase UV-C tolerance. The LD_50_ values for strains GY10973/p11530 (*ssb^+^*/empty expression vector) and GY10973/pSL202 (*ssb^+^*/*ssb* expression plasmid) were equivalent (p>0.01): 744 J•m^−2^ (±41) and 691 J•m^−2^ (±70), respectively (Figure 5). However, there was a 100-fold reduction in survival of SL102 (Δ*ssb/ssb* expression plasmid) between 200 J•m^−2^ and 400 J•m^−2^, and further 10-fold reductions at 600 J•m^−2^ and 800 J•m^−2^_,_ at which point no surviving colonies were visible in the 10^−1^ dilution spot. GY10973/p11530 and GY10973/pSL202 demonstrated only 10-fold reduction in survival at 800 J•m^−2^. Additionally, the LD_50_ of SL102 was 231 J•m^−2^ (±6), which is 33.4% of the GY10973/pSL202 LD_50_ of 691 J•m^−2^ (±70) (p<0.01). As with ionizing radiation, the expression of *ssb* from the plasmid appeared insufficient to provide wild type levels of resistance to UV.

### *ddrB* cannot Complement the Loss of *ssb*

The genome of *D. radiodurans* is unusual in containing a gene for a radiation-induced, pentameric single-stranded DNA binding protein, DdrB, in addition to the essential, canonical Ssb. The extreme radiation- resistance of *D. radiodurans* is dependent on the presence of both proteins (this study) [17]. To determine if DdrB could substitute for Ssb in terms of growth functions, we attempted to create an *ssb* deletion in a strain carrying *ddrB* on an expression plasmid. Although strain GY10973 has intact *ddrB*, *ddrB* is normally expressed at very low levels in the absence of radiation [17], so transcription from the native promoter may have been insufficient to complement the loss of *ssb* in the previous experiments. To determine if higher expression of *ddrB* could complement loss of *ssb* for growth, strain GY10973/pSL201 (*ddrB* ^+^/*ddrB* expression plasmid) was transformed with the *ssb* replacement fragment as above, and grown in the presence of 0.2 mM IPTG (this level allowed sufficient induction for complementation of *ssb* with pSL202, and allowed equivalent growth to 1 mM IPTG induction; data not shown). If complementation of the *ssb* knockout by the induction of the plasmid-borne *ddrB* was possible, we expected that approximately 8 of 10 transformants (determined from previous *ssb* knockout results) would grow in the presence of IPTG but fail to grow in its absence. Using a statistical analysis similar to that used to determine the essentiality of *ssb*, twenty transformant colonies were randomly picked and replica-plated onto plates containing 0.2 mM IPTG and plates containing no IPTG. All 20 transformants grew on both sets of plates, indicating that they were not *ssb* knockouts, but were heterozygous for *ssb,* containing at least one copy of the intact *ssb* gene within the multiple genome copies. This demonstrated that the increased *ddrB* transcript resulting from our expression system, and thus increased DdrB, cannot complement the loss of Ssb (p<.001). This same expression plasmid provides complementation to a *ddrB* deletion (see below), verifying the production of functional protein. These results indicate that the two proteins likely have significantly different roles in the general DNA metabolism of *D. radiodurans.*

### *ssb* cannot Complement the Loss of *ddrB*

Deletions of *ddrB* result in moderate radiation sensitivity. It was possible that, if Ssb and DdrB had overlapping functions, the presence of the intact *ssb* gene in the chromosome was compensating to some degree. To determine if providing additional Ssb from the plasmid would complement loss of *ddrB*, we created a *ddrB* deletion in strain GY10973 (SL101). Since *ddrB* deletion mutations are viable, but exhibit moderate radiation-sensitivity [17], we tested whether additional Ssb would complement the radiation-sensitive phenotype of strain SL101. SL101 was transformed separately with empty vector p11530, and the expression plasmids pSL201 (carrying *ddrB*) and pSL202 (carrying *ssb*). These strains were irradiated as described above, and survival determined.

**Figure 6:**
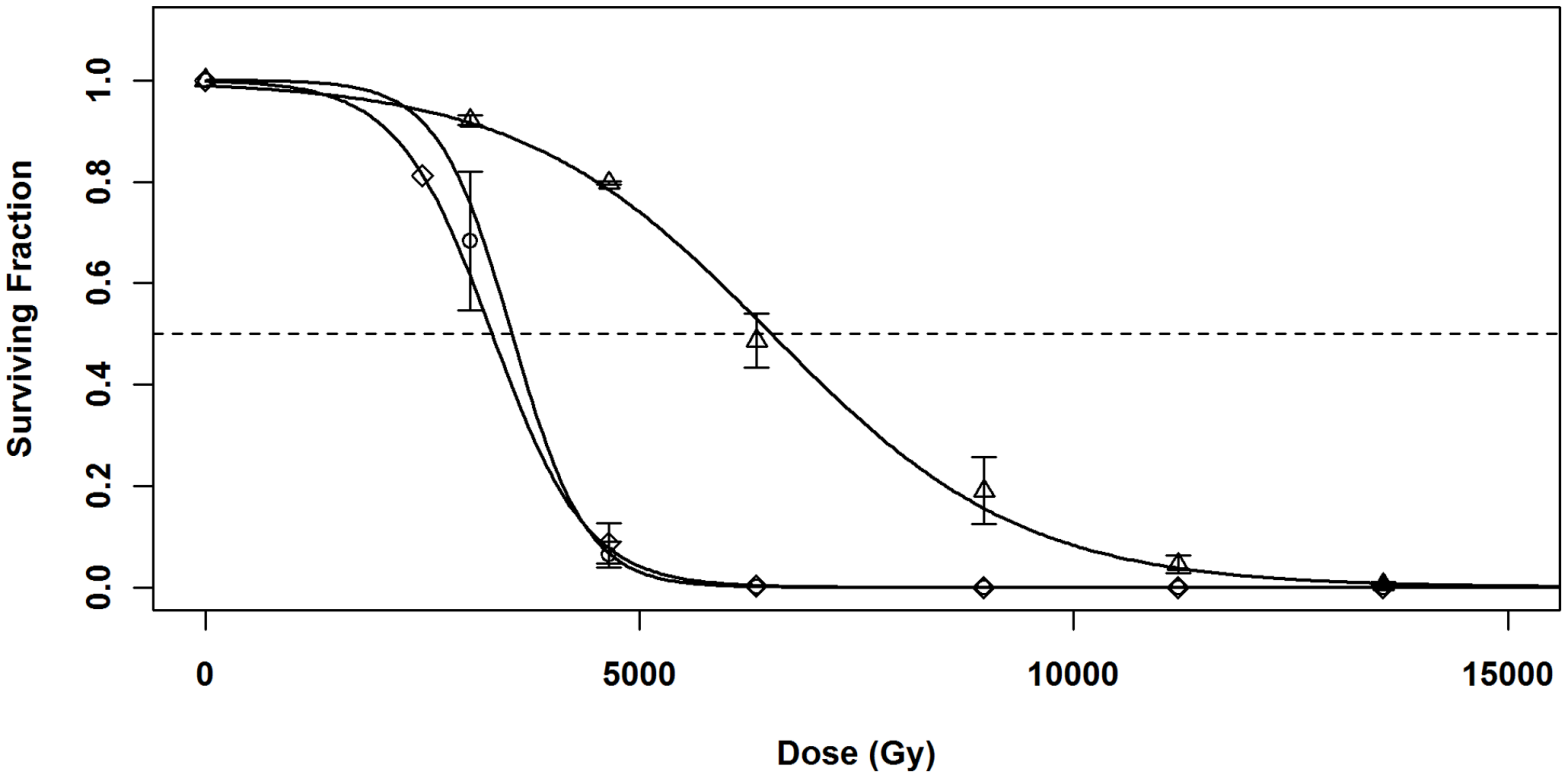
Ionizing radiation survival of *ddrB* knockout strains. Cultures were grown in 1 mM IPTG and irradiated with 23 MeV electrons at a dose rate of 30 Gy/s. (○) SL101/p11530 (Δ*ddrB*/empty expression vector); (Δ) SL101/pSL201 (Δ*ddrB*/*ddrB* expression plasmid); and (◊) SL101/pSL202 (Δ*ddrB*/*ssb* expression plasmid). Dilutions were plated, and survivors counted after 3–5 days. The dashed line indicates 50% survival. Error bars indicate ± standard error.

When strains were grown under full induction (1 mM IPTG), the LD_50_ values for SL101/p11530 (chromosomal Δ*ddrB*/empty vector) and SL101/pSL202 (chromosomal Δ*ddrB*/*ssb* expression plasmid) were 3302±9 Gy and 3530±25 Gy, respectively (Figure 6). These values were statistically equivalent to the LD_50_ value of the parent strain SL101 (p>0.01), and consistent with the phenotype of Δ*ddrB* reported previously [17]. The LD_50_ value for SL101/pSL201 (chromosomal Δ*ddrB*/*ddrB* expression plasmid) was 6520±99 Gy, a significant 185% improvement in radiation tolerance. Thus, complementation of Δ*ddrB in trans* with *ddrB* using this expression system restored radiation tolerance, though not to completely wild-type levels (10750 Gy ±207; Figure 4). However, complementation *in trans* with *ssb* had no effect on radiation tolerance. Once again, this indicates that the two gene products have significantly different roles in *D. radiodurans.*

## Discussion

Single-stranded DNA binding proteins are ubiquitous and highly conserved proteins that protect single-stranded DNA exposed during replication, repair and recombination. In addition to this protective role, SSBs bind heterologous proteins and act as coordinators of these multiple processes [5]. Their roles in all aspects of DNA metabolism renders them essential proteins in all cells where a single homolog exists. In *D. radiodurans,* a second, unique pentameric single-stranded DNA binding protein (encoded by *ddrB*) has recently been identified as highly up-regulated in response to radiation and is thought to play a major role in the radiation resistance of this organism. In support of this, deletions of this gene are viable, but render the cells moderately radiation sensitive [17]. The canonical SSB, encoded by *ssb*, has been assumed to play a major role in DNA replication, and deletions would therefore be inviable. Ssb levels are also regulated at the transcriptional level by radiation [13], but the essentiality of this gene has never been demonstrated. In this study, we examined the effects of altered SSB gene expression, specifically the canonical-type bacterial SSB encoded by *ssb*, on growth and radiation tolerance in the bacterium *D. radiodurans*. We demonstrated that this gene is essential for growth under normal conditions. The lethality of a deletion in *ssb* cannot be compensated by constitutive expression of *ddrB*. Consistent with this, the radiation-sensitive phenotype of Δ*ddrb* cannot be suppressed by constitutively providing *ssb in trans* at levels that suppress the growth phenotype of an *ssb* deletion.

In strain SL102, lacking a chromosomal copy of *ssb,* but complemented with *ssb in trans* under control of a chemically induced promoter, the ionizing radiation LD_50_ was reduced to less than 10% of wild type levels. This effectively brought the radiation tolerance of *D. radiodurans* into the realm of the radiation-sensitive bacteria, such as *E. coli*. The reduction correlates well with the *ssb* expression levels provided from the inducible expression plasmid. Under unstressed conditions, with the expression plasmid fully induced, SL102 only expressed *ssb* transcript at 42% of wild-type levels. It is important to note that although expression of *ssb* is elevated in response to damage in wild-type cells, with this system transcript levels cannot be adjusted by the cell in response to DNA damage, since expression is controlled solely through chemical induction of the promoter. The significance of this characteristic is highlighted when considering the situation in which protein production is directly proportional to gene expression, with no significant translational regulation. This correlation has been demonstrated for *D. radiodurans* Ssb, where exposure to radiation resulted in a 6-8-fold increase in transcription of *ssb* and a 6-fold increase in Ssb protein levels [13]. In our system, after irradiation, Ssb levels in SL102, in which expression is not affected by radiation, would be only 7%–10% of wild-type levels. This correlates well with the observed ionizing radiation LD_50_ value of SL102 at approximately 7% of wild-type. Previous reports have shown that UV radiation does not promote an increase in *ssb* transcription in *D. radiodurans* [13]. The UV radiation LD_50_ value was approximately 33% of wild-type, which correlates closely with our measured *ssb* expression at 42% of wild-type. Though these numbers are based on a simplified model, it provides a solid basis for the hypothesis that expression of *ssb* can essentially “make or break” the extreme natural radiation tolerance of *D. radiodurans.*

It is interesting to note that though ultraviolet and ionizing radiation produce different types of DNA damage, tolerances to both were significantly impacted by reduced *ssb* expression. Furthermore, reduction in tolerances correlated closely with predicted reductions in *ssb* gene expression. These results highlight the centrality of Ssb to all DNA repair pathways, regardless of the type of damage involved. Additionally, this further supports the role of Ssb and its levels as a wide-range determinant of DNA damage tolerance within the cell.

In addition to dramatically affecting radiation tolerance, *ssb* expression significantly influenced growth capabilities of the cell, but at levels far below those necessary for extreme resistance. We demonstrated that growth was dependent on *ssb* expression. In particular, we have shown that *ssb* expression is essential for cell survival. Further, *ssb* expression at 42% of unstressed wild-type levels, although sufficient to support normal growth, resulted in extreme radiation sensitivity. This suggests that higher concentrations of *ssb* are necessary for DNA repair pathways than replication pathways. Moreover, reducing *ssb* expression to 28.5% of wild-type levels hindered growth capabilities, but was not lethal. Thus, the threshold for unhindered growth is likely somewhere between these expression levels (28–42% of unstressed, wild type). Based on previous measurements of Ssb protein levels, this correlates to approximately 5500–8000 Ssb dimers per cell [11]. Together these results indicate that *ssb* expression level is not only an important determinant for radiation tolerance, but also for general growth capabilities.

Dramatic changes in the radiation tolerance of *D. radiodurans* were observed by reducing *ssb* expression levels. However, providing additional Ssb over wild-type levels using a plasmid expression system failed to increase radiation tolerance. Addressing this, transcript analysis revealed that there was an insignificant difference in *ssb* transcript levels between strains carrying the fully induced expression plasmid or the empty vector, provided *ssb* on the chromosome was intact. However, in the absence of the chromosomal *ssb*, where only the induced plasmid copy of *ssb* was present, transcript levels were 42% of wild type, indicating that expression should have been significantly increased over wild type in the presence of both genes. The reasons for this are unknown, but perhaps chromosomal expression was down-regulated in response to increased expression from the plasmid, essentially holding the cell at a constant Ssb expression level. Additionally, *ssb* is highly expressed in the wild-type cell at all times and the Ssb concentration increases significantly post-irradiation [11], [17]. Under irradiated conditions, Ssb may not be a limiting component of DNA repair pathways. As such, even if *ssb* expression could have been increased over naturally occurring radiation-induced levels using the inducible plasmid, it may still have had no effect on DNA repair functions, and consequently radiation tolerance. It remains to be seen whether a significant increase in Ssb could increase radiation resistance in *D. radiodurans*.

The Haloarchaea also possess multiple distinct single-stranded DNA binding protein homologs (archaeal RPAs) and associated RPA binding proteins [34]. In *Haloferax volcanii*, only one of three RPA homologs is essential; however, the essentiality can be suppressed by increased transcription of another, non-essential RPA gene [35]. Based on this, we considered that Ssb and DdrB may carry out distinct, but overlapping, functions in the DNA metabolism of *D. radiodurans* [15]. Our results support a separation of function for Ssb and DdrB, which is not unexpected, given the distinct structures and expression of the two proteins [11], [16], [17], [36]. Our expression system appears to provide far less transcription than occurs from the native *ssb* promoter. We cannot rule out that higher levels of expression of either *ssb* or *ddrB* might allow complementation, but we consider this unlikely.

Upon irradiation of *D. radiodurans*, various forms of Ssb are the first proteins seen, followed by DdrB [36]. The integral role of SSBs in replication in general, and the observation of Ssb-containing foci in *D. radiodurans* immediately after irradiation support the necessity of high levels of Ssb-dependent replication in the unusual radiation resistance of this organism. Reconstitution of the genome following irradiation involves high levels of replication [37], which is supported by induction of *ssb* and dependence of radiation resistance on these induced levels. Based on our results, it is likely that Ssb is involved primarily in replication, both during normal growth and immediately following DNA damage, whereas DdrB is not. Processing of Ssb, following irradiation, may serve to modulate heterologous protein binding to allow Ssb to interact with, and recruit, radiation-specific protein partners, including DdrB [18], [36]. DdrB itself binds at least one unique Deinococcal protein, deletions of which confer a distinct growth phenotype, but no radiation tolerance defect [38]. DdrB stimulates single-stranded DNA annealing, even in the presence of Ssb, indicating a distinct role in the progressive reconstitution of the genome [18], [39].

In the broader scope of understanding radiation tolerance mechanisms, it is clear that expression of cellular single-stranded DNA binding proteins is a key component. However, it should be kept in mind that it is not a single determinant for radiation tolerance and is likely a significant player in a larger cell-wide mechanism consisting of several components including protein protection and other components of DNA repair pathways. Further investigation of the protein-protein interactions among the Deinococcal single-stranded DNA binding proteins and their heterologous partners will clarify the distinct roles of both the replication-specific canonical-type Ssb as well as the unique, pentameric DdrB in radiation-resistance.
